# Outpatient Care Fragmentation and Acute Care Utilization in Veterans Affairs Home-Based Primary Care

**DOI:** 10.1001/jamanetworkopen.2022.30036

**Published:** 2022-09-06

**Authors:** Samuel T. Edwards, Liberty Greene, Camila Chaudhary, Derek Boothroyd, Bruce Kinosian, Donna M. Zulman

**Affiliations:** 1 Section of General Internal Medicine, VA Portland Health Care System, Portland, Oregon; 2 General Internal Medicine and Geriatrics, Oregon Health & Science University, Portland; 3 Center to Improve Veteran Involvement in Care, VA Portland Health Care System, Portland, Oregon; 4 Center for Innovation to Implementation, VA Palo Alto Health Care System, Menlo Park, California; 5 Department of Medicine, Stanford University School of Medicine, Stanford, California; 6 Geriatrics Extended Care Data Analysis Center, Corporal Michael J. Crescenz VA Medical Center, Philadelphia, Pennsylvania; 7 Perelman School of Medicine, Division of Geriatrics, University of Pennsylvania, Philadelphia

## Abstract

**Importance:**

Veterans Affairs (VA) Home-Based Primary Care (HBPC) provides comprehensive, interdisciplinary primary care at home to patients with complex, chronic, disabling disease, but little is known about care fragmentation patterns and consequences among these patients.

**Objective:**

To examine outpatient care fragmentation patterns and subsequent acute care among HBPC-engaged patients at high risk of hospitalization or death.

**Design, Setting, and Participants:**

This retrospective cohort study included VA patients aged at least 65 years who were enrolled in the VA and Medicare, whose risk of hospitalization or death was in the top 10%, and who had at least 4 outpatient visits between October 1, 2013, and September 30, 2014. HBPC engagement was defined as having at least 2 HBPC encounters between July 1, 2014, and September 30, 2014. Data were analyzed from March 2020 to March 2022.

**Exposures:**

Two indices of outpatient care fragmentation: practitioner count and the Usual Provider Continuity Index (UPC), based on VA and non-VA health care use from October 1, 2013, to September 30, 2014. All care delivered by HBPC clinicians was analyzed as coming from a single practitioner.

**Main Outcomes and Measures:**

Emergency department (ED) visits and hospitalizations for ambulatory care sensitive conditions (ACSC) from VA records and Medicare claims from October 1, 2014, to September 30, 2015.

**Results:**

Among 8908 identified HBPC patients, 8606 (96.6%) were male, 1562 (17.5%) were Black, 249 (2.8%) were Hispanic, 6499 (73.0%) were White, 157 (1.8%) were other race or ethnicity, and 441 (5.0%) had unknown race or ethnicity; the mean (SD) age was 80.0 (9.02) years; patients had a mean (SD) of 11.25 (3.87) chronic conditions, and commonly had disabling conditions such as dementia (38.8% [n = 3457]). In adjusted models, a greater number of practitioners was associated with increased odds of an ED visit (adjusted odds ratio [aOR], 1.05 [95% CI, 1.03-1.07]) and hospitalization for an ACSC (aOR, 1.04 [95% CI, 1.02-1.06]), whereas more concentrated care with a higher UPC was associated with reduced odds of these outcomes (highest vs lowest tertile of UPC: aOR for ED visit, 0.77 [95% CI, 0.67-0.88], aOR for ACSC hospitalization, 0.78 [95% CI, 0.68-0.88]).

**Conclusions and Relevance:**

Among patients in HBPC, fragmented care was associated with more ED visits and ACSC hospitalizations. These findings suggest that consolidating or coordinating fragmented care may be a target for reducing preventable acute care.

## Key Points

**Question:**

What is the association between care fragmentation and acute care use among Veterans at high risk of hospitalization or death enrolled in Department of Veterans Affairs Home-Based Primary Care (HBPC)?

**Findings:**

In this cohort study of 8908 patients in HBPC at high risk of hospitalization or death, dispersion of outpatient care across more health care practitioners was associated with increased odds of an emergency department visit or a hospitalization for an ambulatory care sensitive condition, while higher concentration of care with the HBPC team was associated with reduced odds of acute care use.

**Meaning:**

These findings suggest that consolidating and/or coordinating fragmented care among HBPC patients may be associated with a reduction in preventable acute care.

## Teaser

This cohort study investigates outpatient care fragmentation patterns and subsequent acute care among Veterans Affairs Home-Based Primary Care–engaged patients at high risk of hospitalization or death.

## Introduction

Older adults with multimorbidity frequently require care from multiple medical specialties and health care practitioners.^1^ With strong coordination from a high-functioning primary care team, care from multiple practitioners can represent efficient care that maximally takes advantage of the knowledge and skills from a range of specialties. Conversely, without coordination from a primary care team, care from multiple practitioners can represent fragmented care, which can increase the burden for patients,^2,3^ and potentially harm them through redundant or unnecessary treatments.^4^ Among Medicare beneficiaries, measures of care fragmentation are associated with increased emergency department (ED) use and hospitalization.^5,6,7^ In the Department of Veterans Affairs, which has implemented a patient-centered medical home model in primary care nationwide, fragmentation of primary care remains associated with lower ratings of practitioner communication, higher ED use and higher mortality.^8^

Veterans Affairs (VA) Home-Based Primary Care (HBPC) is an intensive primary care program that provides comprehensive longitudinal primary care at home to patients with complex, chronic disabling disease.^9^ Care is delivered by an interdisciplinary team consisting of a primary care practitioner, nurse, psychologist, rehabilitation specialist, pharmacist, social worker, and dietitian. HBPC coordinates care within the interdisciplinary team through regular team meetings, and prior studies have shown HBPC teams have strong team function, clear communication, and a collaborative, nonhierarchical workplace culture.^10,11,12^ Additionally, HBPC care has been associated with fewer hospital admissions, lower costs of care, and less acute care at end of life.^13,14,15,16^ HBPC’s high within-team coordination could be a mechanism through which the program prevents acute care use, but the degree of care fragmentation across practitioners, and the relationship between fragmentation and potentially preventable acute care use such as ED visits or hospitalization for ambulatory care sensitive conditions (ACSC), is unknown.

In this study, we examined fragmentation of care across practitioners among patients at high risk of hospitalization or death enrolled in HBPC and examined the association between care fragmentation measures and acute care utilization. We hypothesized that HBPC teams would provide most of their patients’ outpatient care, but that care fragmentation would remain associated with acute care use. Alternatively, we considered that HBPC may overcome the detrimental effects of fragmentation, and hence care fragmentation measures would not be associated with acute care use.

## Methods

### Study Design and Cohort

We conducted a retrospective cohort study of care continuity patterns and acute care utilization among veterans aged 65 years and older participating in HBPC. The cohort for this analysis was drawn from an ongoing study of care continuity and fragmentation that focused on patients who were at high risk for 1-year hospitalization based on the VA’s Care Assessment Need (CAN) risk score.^17,18^

The study dates were from October 1, 2013, to September 30, 2015. Patient characteristics and care fragmentation scores were constructed using data from October 1, 2013, to September 30, 2014 (hereafter fiscal year 2014 [FY14]), and outcomes were measured from October 1, 2014, to September 30, 2015 (hereafter fiscal year 2015 [FY15]). Patients were included if they were alive on September 30, 2014, and their last recorded CAN score in FY14 was in the top 10th percentile, and they were continuously enrolled in Medicare Fee-for-Service (FFS) Part A and Part B, and not enrolled in Medicare Advantage for the entire study period. Patients were excluded if they had fewer than 4 outpatient visits due to minimal variation in care fragmentation. Patients were considered HBPC-engaged if they had 2 or more HBPC visits, from July 1, 2014, to September 30, 2014 (4th quarter FY14). For all included patients, we analyzed data for VA care (care that took place in VA facilities), VA-purchased care (care in non-VA facilities paid for by VA, hereafter referred to as Community Care), and care in non-VA facilities paid for by Medicare. This study was approved by the VA Palo Alto Health Care System Research & Development Committee and Stanford University institutional review board with a waiver of informed consent due to the impractical nature of obtaining consent from a large number of patients. This report follows the Strengthening the Reporting of Observational Studies in Epidemiology (STROBE) reporting guideline.

### Care Fragmentation Measures

To assess fragmentation in outpatient practitioner visits, we combined health care utilization data from the electronic medical record (VA) and that generated from billing systems (Medicare and Community Care) based on an adaptation of the method described in Burgess et al.^19^ We defined an eligible encounter as a clinic- or home-based visit for evaluation and management, care coordination, or psychotherapy and other mental health services (as defined by *Current Procedural Terminology* and Healthcare Common Procedure Coding System codes). Using practitioner specialty codes, we included medical visits conducted by a physician, nurse practitioner (NP), or physician assistant (PA); for mental health care visits, we included visits with a psychiatrist, psychologist, or licensed clinical social worker. National Provider Index (NPI) was used to identify unique practitioners. HBPC encounters were identified based on HBPC-specific visit codes. Urgent care and ED visits were not considered outpatient care.

Outpatient care fragmentation was calculated in FY14 using 2 measures: the total number of outpatient practitioners seen and the proportion of care with an empirically defined usual practitioner using the Usual Provider Continuity Index (UPC).^20^ A higher number of practitioners seen represents more fragmented care. UPC has a range from 0 to 1, where a higher UPC represents less fragmented care, and a UPC of 1 represents care from only 1 practitioner. To reflect the highly collaborative, integrated nature of HBPC care, all care delivered by HBPC primary care and mental health clinicians (physician, nurse practitioner, physicians assistant, psychologist, psychiatrist, social work) was considered from a single practitioner. We identified the usual practitioner as the clinician who the patient saw most frequently.

### Outcome Measures

Two acute care utilization outcomes were constructed using VA, Community Care and Medicare data for FY15: (1) hospitalizations for ACSCs and (2) ED visits. Hospitalization for ACSCs are hospitalizations for conditions that could potentially be prevented with timely and appropriate ambulatory care. We used the Agency for Healthcare Research and Quality’s (AHRQ) Prevention Quality Indicators (PQI) to define ACSCs.^21,22^ Thirteen ACSC conditions were included: diabetes with short-term or long-term complications, perforated appendix, chronic obstructive pulmonary disease (COPD) or asthma in older adults, hypertension, heart failure, dehydration, bacterial pneumonia, urinary tract infection, angina, uncontrolled diabetes, and lower-extremity amputation (complete definitions in eMethods 1 in the Supplement). Inpatient stays were classified as an ACSC hospitalization based on principal diagnosis and in some cases, secondary diagnoses and procedures. Using previously described algorithms, we identified ED visits regardless of whether they resulted in discharge or hospitalization.^23,24^

### Covariates

Patient-level covariates included demographic characteristics, clinical characteristics, health care utilization, and HBPC visits in FY14. Age, gender, marital status, self-reported race and ethnicity, urban or rural status, and VA enrollment priority (based on military service and income^25^) were derived from VA enrollment data for FY14. Given known differences in acute care use by race and ethnicity that likely arise from systemic racism and differential access to care, we included race and ethnicity as covariates to account for potential confounding in estimating the association between fragmentation measures and acute care use. Number of chronic conditions were identified using *International Classification of Diseases, Ninth Revision (ICD-9)* diagnosis codes for 47 conditions as categorized by the AHRQ and VA’s Women’s Health Evaluation Initiative (eMethods 2 in the Supplement).^26,27^ Conditions were coded as present if they occurred in 2 outpatient or 1 inpatient visit in FY14. The presence of any mental health condition was identified using 10 mental health–related chronic conditions (eMethods 2 in the Supplement). We also calculated the total number of visits across VA and Medicare, an indicator for any Medicare encounter, and the number of total HBPC visits to all HBPC team members (physicians, nurse practitioners, physician assistants, psychologists, social workers, nurses, dietitians, pharmacists, and rehabilitation therapists) in the last quarter of FY14.

### Statistical Analysis

We fit 2 logistic regression models to test the association between HBPC-constructed care fragmentation in FY14 and (1) ACSC hospitalization in FY15 and (2) any ED visit in FY15. Analyses were adjusted for demographic characteristics, clinical characteristics, health care utilization and number of HBPC visits in FY14 as noted under covariates. We modeled practitioner count as a continuous measure and UPC in tertiles.

To address the competing risk of death, we also ran models excluding those deceased in the follow-up year as sensitivity analyses. To address concerns about the biasing effect of those with perfect continuity (ie, zero care fragmentation, UPC = 1), we also ran models excluding those with a UPC of 1. Based on the distribution of the number of ED visits observed in the cohort, we ran a final sensitivity analysis modeling the number of ED visits as a count variable, using a negative binomial distribution. To ensure our team-based definition of HBPC practitioners did not bias our results, we also performed regression analyses with care fragmentation measures calculated not considering multiple HBPC practitioners as a team.

We used SAS statistical software version 9.4 (SAS Institute) to construct the data set and Stata statistical software version 15 (StataCorp) to conduct statistical analyses. We used a 2-sided *P* < .05 as the significance threshold. Data were analyzed from March 2020 to March 2022.

## Results

### Patient Characteristics

**Figure 1.**
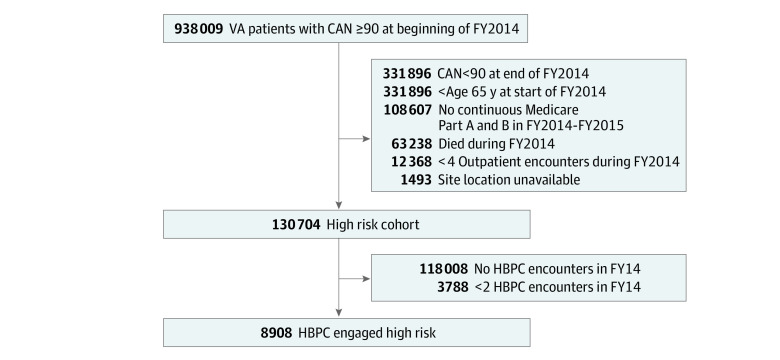
Flow Diagram of Analytic Cohort Abbreviations: CAN, Care Assessment Needs Score; FY2014, fiscal year 2014; FY2015, fiscal year 2015; HBPC, Home-Based Primary Care; VA, Veterans Affairs.

We identified 130 704 VA patients at high risk of hospitalization or death in FY14 (subsequently referred to as high-risk patients), 8908 of whom were engaged in HBPC at the end of FY14 (Figure 1). Detailed patient characteristics are listed in Table 1. Among 8908 identified HBPC patients, 8606 (96.6%) were male, 1562 (17.5%) were Black, 249 (2.8%) were Hispanic, 6499 (73.0%) were White, 157 (1.8%) were other race or ethnicity, and 441 (5.0%) had unknown race or ethnicity; the mean age was 80.0 (9.0) years, with 3177 patients (35.7%) aged at least 85 years; patients had a mean (SD) of 11.25 (3.87) chronic conditions, such as heart failure (49.7% [n = 4431]), diabetes (54.3% [n = 4836]), dementia (38.8% [n = 3457]), or a mental health condition (61.0% [n = 5437]); 6095 patients (68.4%) lived in an urban setting; and 5979 (67.0%) were in VA priority groups for patients with high disability (priority groups 1 and 4). HBPC patients had an estimated probability of hospitalization in 1 year of 41%, and 1609 HBPC patients (18.1%) were in the top 1% of estimated hospitalization risk. When compared with other high-risk patients, HBPC patients were older, had more chronic conditions, and a higher estimated risk of hospitalization (eTable 1 in the Supplement).

**Table 1.** Patient Characteristics and Care Fragmentation Among HBPC Patients at High Risk of Hospitalization

Characteristic	HBPC-engaged high-risk patients, No. (%)
No.	8908
CAN 1 y hospitalization probability^a^	0.41 (0.17)
CAN percentiles	
90th	3375 (37.9)
95th	798 (9.0)
96th	891 (10.0)
97th	986 (11.1)
98th	1249 (14.0)
99th	1609 (18.1)
Gender	
Female	302 (3.4)
Male	8606 (96.6)
Age, mean (SD), y	80.0 (9.0)
Marital status	
Not married	5091 (57.2)
Married	3761 (42.2)
Missing	56 (0.6)
VA priority group	
7 + 8	406 (4.6)
5 (low income)	1482 (16.6)
1 + 4 (high disability)	5970 (67.0)
2 + 3 + 6 (low/moderate disability)	1050 (11.8)
Race and ethnicity	
Black	1562 (17.5)
Hispanic	249 (2.8)
White	6499 (73.0)
Other	157 (1.8)
Unknown	441 (5.0)
Urban or rural	
Urban	6095 (68.4)
Rural or highly rural	2813 (31.6)
No. chronic conditions, mean (SD)	11.25 (3.87)
Any mental health condition	5437 (61.0)
Selected chronic conditions	
Hypertension	8174 (91.8)
Coronary artery disease	4973 (55.8)
Heart failure	4431 (49.7)
Diabetes	4836 (54.3)
Depression	4029 (45.2)
Posttraumatic stress disorder	1246 (14.0)
Anxiety disorders	1784 (20.0)
Dementia	3457 (38.8)
Renal failure or nephropathy	4101 (46.0)
Chronic obstructive pulmonary disease	4165 (46.8)
Care fragmentation measures	
No. of practitioners^b^	
Mean (SD)	5.65 (3.55)
Median (IQR)	5.00 (3.00-7.00)
UPC^c^	
Mean (SD)	0.55 (0.24)
Median (IQR)	0.50 (0.33-0.75)

Abbreviations: CAN, Care Assessment Need; HBPC, Home-Based Primary Care; UPC, Usual Provider Continuity Index; VA, Veterans Affairs.

^a^ Care Assessment and Needs Score.

^b^ Higher numbers of practitioners indicate higher care fragmentation.

^c^ UPC, the proportion of care with the most frequently seen practitioner, ranges from 0 to 1 where 1 indicates that all visits occur with one practitioner. Lower UPC indicates higher care fragmentation.

### Baseline Health Care Use

HBPC patients had a mean (SD) of 15.4 (9.3) outpatient practitioner visits in FY14, and 3047 (34.2%) had any Medicare claim for an outpatient visit. Examining the full range of visits delivered by HBPC team members including physicians, nurse practitioners, physician assistants, psychologists, social workers, nurses, dieticians, pharmacists and therapists, HBPC patients had a mean (SD) of 37.0 (22.19) visits in FY14, and a mean (SD) of 11.1 (SD 6.5) visits to any HBPC team member during Q4 FY14.

### Care Fragmentation Measures

**Figure 2.**
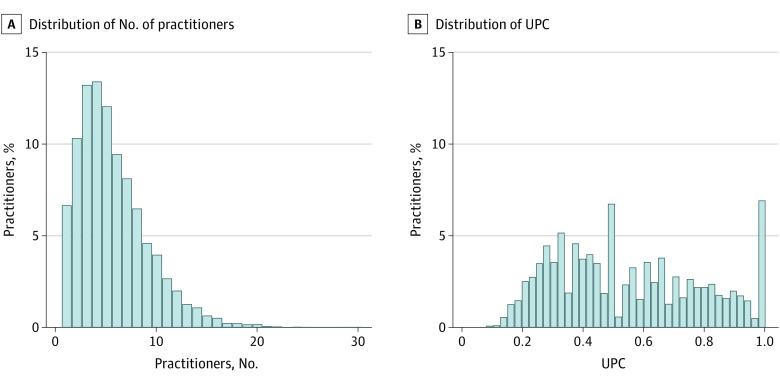
Distribution of Practitioner Count and Usual Provider Continuity Index (UPC) for Patients at High Risk of Hospitalization Receiving Home-Based Primary Care

HBPC patients saw a mean of 5.65 practitioners (range, 1-30 practitioners) in FY14. The mean (SD) UPC for HBPC patients was 0.55 (0.24), indicating that for HBPC patients, a mean of 55% of outpatient care was provided by the HBPC team (Table 1, Figure 2). For 608 HBPC patients (6.8%), both the practitioner count and the UPC was 1.0, indicating that the HBPC team provided all outpatient care during FY14. When compared with non–HBPC high-risk patients, HBPC patients saw fewer practitioners and UPC was higher (eTable 1 in the Supplement). Patient characteristics stratified by UPC tertiles are presented in eTable 2 in the Supplement. Patients in the highest tertile of UPC (least fragmented) were older (mean [SD] age in highest UPC tertile: 81.49 [9.04] years vs lowest tertile UPC: 78.75 [8.87] years; *P* < .001) and had fewer chronic conditions (mean [SD] number of chronic conditions among highest UPC tertile: 10.62 [3.68] vs lowest tertile UPC: 11.73 [SD 3.96]; *P* < .001).

### Outcomes

Table 2 presents clinical outcomes in FY15. Among 8908 HBPC patients, 6506 (73.0%) had at least 1 ED visit, and the mean (SD) number of ED visits was 2.33 (2.80); 5107 (57.3%) were hospitalized at least once; 1597 (22.0%) had a hospitalization for an ACSC; and 2400 (26.9%) died in FY15.

**Table 2.** Clinical Outcomes Among HBPC Patients at High Risk of Hospitalization During FY15

Outcome	HBPC-engaged high-risk patients, No. (%) (N = 8908)
Hospitalization	5107 (57.3)
Deceased	2400 (26.9)
Hospitalized or died	5757 (64.6)
ACSC hospitalization	1957 (22.0)
Any ED visit	6506 (73.0)
ED visits, mean (SD)	2.33 (2.80)

Abbreviations: ACSC, Ambulatory Care Sensitive Condition; ED, emergency department; FY15, fiscal year 2015.

Table 3 presents the association between outpatient care fragmentation and acute care use for patients in HBPC. After adjusting for patient characteristics and health care use during FY14, each additional practitioner involved in an HBPC patient’s care (representing more fragmented care) was associated with 1.05 increased odds of an ED visit (95% CI, 1.03-1.07) and a 1.04 increased odds of hospitalization for an ACSC (95% CI, 1.02-1.06). When care was more concentrated with the HBPC team (represented by a higher UPC index), patients were less likely to experience an ED visit (highest tertile of UPC vs lowest tertile aOR for ED visit: 0.77 [95% CI, 0.67-0.88]) and a hospitalization for an ACSC (highest tertile of UPC vs lowest tertile aOR for ACSC hospitalization: 0.78 [95% CI, 0.68-0.88]). Full regression output is presented in eTable 3 in the Supplement. Sensitivity analyses that excluded deceased patients, excluded patients with UPC = 1, modeled ED visits as a count variable, and that did not consider HBPC teams as a single practitioner, revealed similar associations between outpatient care fragmentation and outcomes (eTables 4-7 in the Supplement).

**Table 3.** Adjusted Association Between Outpatient Care Fragmentation in FY14 and FY15 Hospitalization or ED Visit for HBPC Patients at High Risk of Hospitalization

Outcome	Fragmentation measure	OR (95% CI)^a^
Any ACSC hospitalization	Practitioner count^b^	1.04 (1.02-1.06)
	UPC: low^c^	1 [Reference]
	UPC: medium^c^	0.94 (0.83-1.06)
	UPC: high^c^	0.77 (0.67-0.88)
Any ED visit	Practitioner count^b^	1.05 (1.03-1.07)
	UPC: low^c^	1 [Reference]
	UPC: medium^c^	0.85 (0.76-0.96)
	UPC: high^c^	0.78 (0.68-0.88)

Abbreviations: ACSC, Ambulatory Care Sensitive Condition; ED, emergency department; FY14, fiscal year 2014; FY15, fiscal year 2015; HBPC, Home-Based Primary Care; OR, odds ratio; UPC, Usual Provider Continuity Index.

^a^ Models adjusted for demographics and clinical characteristics. N = 8852.

^b^ Higher practitioner count represents more fragmentation.

^c^ Higher UPC (closer to 1) represents more concentrated (less fragmented) care; tertiles: low, 0.08 to 0.40; medium, more than 0.40 to 0.67; high, more than 0.67 to 1.

## Discussion

In this retrospective cohort study of outpatient care fragmentation and acute care utilization among high-risk HBPC patients, we found that more than half of outpatient care was delivered by the HBPC team, but there remained a range of care fragmentation across practitioners. Patients with more fragmented outpatient care experienced more ED visits and hospitalizations for ACSCs in the subsequent year. Although HBPC has a low level of fragmentation given the complexity of the patients it cares for, the persistent association of care fragmentation with acute care outcomes suggests that addressing outpatient care fragmentation may present an opportunity to reduce acute health care use.

The association that we observed between care fragmentation and acute care use aligns with studies of high-risk patients in other contexts. Nyweide et al^7^ demonstrated that among Medicare beneficiaries, care continuity is associated with fewer ACSC hospitalizations, while Kern et al^4,6^ demonstrated care fragmentation was associated with increased ED visits among Medicaid beneficiaries in New York, and that this association varied by medical complexity. Similar findings were observed when the VA implemented a team-based patient-centered medical home model,^28,29,30^ but in other settings, care fragmentation measures have not been found to be associated with acute care outcomes, or have been associated with lower rates of hospitalization for ACSCs.^18^ Our work contributes to literature demonstrating that in VA HBPC patients at high risk of hospitalization, there is an association between care fragmentation and negative clinical outcomes.^30^

Our work has important policy implications. Through the passage of the 2014 Veterans Access, Choice, and Accountability Act (Choice Act)^31^ and the 2018 Maintaining Internal Systems and Strengthening Integrated Outside Networks (MISSION) Act,^32^ the VA has committed to referring veterans to non-VA health care practitioners to increase access to care, and in 2021, the VA allocated nearly one-quarter of its health care budget to purchasing non–VA care.^33^ Our findings suggest that among patients at high risk of hospitalization in HBPC, increased outpatient fragmentation, such as the dispersion of care caused by referral to non-VA practitioners, could be associated with greater acute care use.

HBPC patients receive relatively low levels of fragmented care, given their level of medical complexity. As HBPC teams comprise multiple disciplines and develop expertise in comprehensive care for complex chronically ill patients, it is intuitive that teams may be able to address most care needs internally, and refer less to other practitioners. For example, the integration of mental health practitioners may obviate the need to refer to a psychiatrist or counselor, reducing fragmentation. Additionally, HBPC team members’ strong in-team relationships may lead to fewer visits and less fragmented care. Frequent in-person interdisciplinary team meetings, informal in-person communication and use of cell phones allow practitioners to rapidly consult each other to solve clinical issues, preventing referral to outside practitioners. We also found a subgroup of patients for whom HBPC provided 100% of their outpatient care. This could represent individuals who are completely homebound, or for whom HBPC has developed sufficient expertise such that they require no input from specialists. Alternatively, these patients may have reached a level of clinical stability or disease progression where specialist, curative care has less to offer.

Less care fragmentation in HBPC may lead to less acute care use by multiple mechanisms, beyond responsive, trusted primary care, and advanced illness programs such as hospital at home, providing acute care in the home setting.^33,34^ The presence of a pharmacist on the HBPC team may identify polypharmacy and harmful drug interactions, reducing fragmented prescribing from multiple practitioners, and reduce related acute care use.^34^ The team may perform a bridging function, consulting with specialist colleagues and delivering care to the patient without requiring visits to multiple practitioners. The interdisciplinary team may perform an integrating function, ensuring that multiple other practitioners have the same salient information, and ensuring that care is adherent to patient goals, a function particularly important for practitioners outside the VA ecosystem.

### Limitations

This study has some limitations. First, to examine care fragmentation in HBPC, we constructed care fragmentation measures that included multiple practitioner types on the HBPC team, which was not possible for non–HBPC practitioners. However, based on our understanding of the HBPC clinical model, this team-based measure is an accurate description of the nature of HBPC care,^11^ and other studies have considered multiple practitioners as a group for measurement purposes.^35^ Second, we defined HBPC engagement using HBPC visit codes, which may overestimate HBPC enrollment. However, this has been done in prior studies.^14,15^ Third, we examined outcomes at a specific time interval using logistic regression, which can be problematic with a group experiencing a high rate of ED use, hospitalization, and mortality. Future work could include a time to event analysis with competing risks. Fourth, we did not include patients enrolled in Medicare Advantage, and as such, our study may not generalize to this group. Fifth, we treated HBPC as a standardized national program and did not examine regional variation in care fragmentation, but this could be an area for further work. Finally, care fragmentation measures could be endogenous to medical complexity, and while we adjusted for number of chronic conditions, our observed associations between fragmentation, acute care use could in part be related to residual confounding. Further work could explore the association between medical complexity and care fragmentation.

## Conclusions

Among a medically complex population of patients receiving HBPC, fragmented care was associated with more ED visits and ACSC hospitalizations. These findings suggest that further consolidating or coordinating fragmented care may be beneficial in this population, and care patterns might be a potential target for reducing preventable acute care.

## Notes

Supplement. **eMethods 1.** Definitions of Ambulatory Care Sensitive Conditions (ACSC) from the Agency for Healthcare Research and Quality’s (AHRQ) Prevention Quality Indicators (PQI) **eMethods 2.** AHRQ Chronic Conditions **eTable 1.** Patient Characteristics, Care Fragmentation Scores and Outcomes for Overall High-Risk Patient Cohort (n=130, 704) **eTable 2.** Patient Characteristics by UPC Tertile **eTable 3.** Full Logistic Regression Models of Relationship Between Outpatient Care Fragmentation in FY14 and FY15 Hospitalization or ED Visit for HBPC Patients (n=8,852) **eTable 4.** Sensitivity Analysis: Full Logistic Regression Models of Relationship Between Outpatient Care Fragmentation in FY14 and FY15 Hospitalization or ED Visit for HBPC Patients, Excluding Those Who Died in FY15 (n=6,464) **eTable 5.** Sensitivity Analysis: Negative Binomial Regression Models of Relationship Between Outpatient Care Fragmentation in FY14 and Number of FY15 ED Visits for HBPC Patients (n=8,852) **eTable 6.** Sensitivity Analysis: Adjusted Relationship Between UPC in FY14 and FY15 Hospitalization or ED Visit for HBPC Patients, Excluding Those With UPC = 1 (n=8,247) **eTable 7.** Adjusted Relationship Between Outpatient Care Fragmentation in FY14 and FY15 Hospitalization or ED Visit for HBPC Patients, All Practitioners Considered Unique (No Accounting for HBPC Team)
