# Supplementary material for: Outpatient Care Fragmentation and Acute Care Utilization in Veterans Affairs Home-Based Primary Care
Source: JAMA Netw Open. 2022 Sep 6;5(9):e2230036. doi: 10.1001/jamanetworkopen.2022.30036 (PMC9449785; doi:10.1001/jamanetworkopen.2022.30036)
Supplement: Supplement. — eMethods 1. Definitions of Ambulatory Care Sensitive Conditions (ACSC) from the Agency for Healthcare Research and Quality’s (AHRQ) Prevention Quality Indicators (PQI) eMethods 2. AHRQ Chronic Conditions eTable 1. Patient Characteristics, Care Fragmentation Scores and Outcomes for Overall High-Risk Patient Cohort (n=130, 704) eTable 2. Patient Characteristics by UPC Tertile eTable 3. Full Logistic Regression Models of Relationship Between Outpatient Care Fragmentation in FY14 and FY15 Hospitalization or ED Visit for HBPC Patients (n=8,852) eTable 4. Sensitivity Analysis: Full Logistic Regression Models of Relationship Between Outpatient Care Fragmentation in FY14 and FY15 Hospitalization or ED Visit for HBPC Patients, Excluding Those Who Died in FY15 (n=6,464) eTable 5. Sensitivity Analysis: Negative Binomial Regression Models of Relationship Between Outpatient Care Fragmentation in FY14 and Number of FY15 ED Visits for HBPC Patients (n=8,852) eTable 6. Sensitivity Analysis: Adjusted Relationship Between UPC in FY14 and FY15 Hospitalization or ED Visit for HBPC Patients, Excluding Those With UPC = 1 (n=8,247) eTable 7. Adjusted Relationship Between Outpatient Care Fragmentation in FY14 and FY15 Hospitalization or ED Visit for HBPC Patients, All Practitioners Considered Unique (No Accounting for HBPC Team) [file jamanetwopen-e2230036-s001.pdf]

# Supplemental Online Content

Edwards ST, Greene L, Chaudhary C, Boothroyd D, Kinosian B, Zulman DM. Outpatient care fragmentation and acute care utilization in Veterans Affairs Home-Based Primary Care. *JAMA Netw Open.* 2022;5(9):e2230036. doi:10.1001/jamanetworkopen.2022.30036

**eMethods 1.** Definitions of Ambulatory Care Sensitive Conditions (ACSC) from the Agency for Healthcare Research and Quality's (AHRQ) Prevention Quality Indicators (PQI)

**eMethods 2.** AHRQ Chronic Conditions

**eTable 1.** Patient Characteristics, Care Fragmentation Scores and Outcomes for Overall High-Risk Patient Cohort (n=130, 704)

**eTable 2.** Patient Characteristics by UPC Tertile

**eTable 3.** Full Logistic Regression Models of Relationship Between Outpatient Care Fragmentation in FY14 and FY15 Hospitalization or ED Visit for HBPC Patients (n=8,852)

**eTable 4.** Sensitivity Analysis: Full Logistic Regression Models of Relationship Between Outpatient Care Fragmentation in FY14 and FY15 Hospitalization or ED Visit for HBPC Patients, Excluding Those Who Died in FY15 (n=6,464)

**eTable 5.** Sensitivity Analysis: Negative Binomial Regression Models of Relationship Between Outpatient Care Fragmentation in FY14 and Number of FY15 ED Visits for HBPC Patients (n=8,852)

**eTable 6.** Sensitivity Analysis: Adjusted Relationship Between UPC in FY14 and FY15 Hospitalization or ED Visit for HBPC Patients, Excluding Those With UPC = 1 (n=8,247)

**eTable 7.** Adjusted Relationship Between Outpatient Care Fragmentation in FY14 and FY15 Hospitalization or ED Visit for HBPC Patients, All Providers Considered Unique (No Accounting for HBPC Team)

This supplemental material has been provided by the authors to give readers additional information about their work.

© 2022 Edwards ST et al. *JAMA Network Open.*

**eMethods 1. Definitions of Ambulatory Care Sensitive Conditions (ACSC) from the Agency for Healthcare Research and Quality's (AHRQ) Prevention Quality Indicators (PQI)**

| **PQI** | **Defining Principal Diagnosis** |
|---|---|
| Diabetes with short-term complications | 25010, 25011, 25012, 25013, 25020, 25021, 25022, 25023, 25030, 25031, 25032, 25033 |
| Perforated appendix | 5400, 5401 |
| Diabetes long term complications | 25040,  25041, 25042, 25043, 25050, 25051, 25052, 25053, 25060, 25061, 25062, 25063, 25070, 25071, 25072, 25073, 25080, 25081, 25082, 25083, 25090, 25091, 25092, 25093 |
| COPD/asthma in older adults[1, 3] | 490, 4660, 494, 496, 4910, 4911, 4918, 4919, 4920, 4928, 4940, 4941, 49120, 49121, 49122 |
| Hypertension[2, 3] | 4010, 4019, 40200, 40210, 40290, 40300, 40310, 40390, 40400, 40410, 40490 |
| Heart Failure[2] | 4280, 4281, 4289, 39891, 40201, 40211, 40291, 40401, 40403, 40411, 40413, 40491, 40493, 42820, 42821, 42822, 42823, 42830, 42831, 42832, 42833, 42840, 42841, 42842, 42843 |
| Dehydration[1] | 27650, 27651, 27652 |
| Bacterial Pneumonia[2, 3] | 481, 485, 486, 4822, 4829, 4830, 4831, 4838, 48241, 48230, 48231, 48232, 48239, 48242 |
| Urinary Tract Infection[2, 3] | 5902, 5903, 5909, 5950, 5959, 5990, ,59010, 59011, 59080, 59081 |
| Angina[2] | 4111, 4130, 4131, 4139, 41181, 41189 |
| Uncontrolled Diabetes | 25002, 25003 |
| Lower-extremity amputation in adults with diabetes[, 2, 3] | Procedure codes:  8410, 8412, 8413, 8414, 8415, 8416, 8417, 8418, 8419<br>With Diagnoses codes from above |

[1]Secondary diagnosis code pairing required
[2]Exclusion procedures apply
[3]Exclusion diagnoses apply

© 2022 Edwards ST et al. *JAMA Network Open.*

**eMethods 2: AHRQ Chronic Conditions**

| Category | Condition |
|---|---|
| Cancer | Cancer |
| Cardiovascular | Arrhythmia/Conduction Disorder |
| | Coronary Artery Disease |
| | Cerebrovascular Disease |
| | Heart Failure |
| | Hypertension |
| | Valvular Disease |
| | Vascular Disease |
| Endocrine/Metabolic/Nutritional | Diabetes Mellitus |
| | Lipid Disorders |
| | Overweight/Obesity |
| | Osteoporosis |
| | Thyroid disorders |
| Gastrointestinal | Esophageal/Gastric/Duodenal Disorders |
| | Liver Disease or Hepatitis C |
| | Inflammatory Bowel Disease |
| Hematologic/Immunologic | Anemia |
| | Coagulation and Hemorrhagic Disorders |
| | Thrombocytopenia |
| Infectious Disease | HIV/AIDS |
| | Tuberculosis |
| Mental Health/Substance Use Disorder (SUD) | Alcohol Use Disorders |
| | Anxiety Disorders - Other |
| | Bipolar Disorders |
| | Depression |
| | Drug Use Disorders – non opioid |
| | Opioid Drug Use Disorders |
| | Personality Disorders |
| | Psychotic Disorders - Other |
| | PTSD |
| | Schizophrenia |
| Musculoskeletal | Joint Disorders |
| | Rheumatologic/Autoimmune Disorders |
| | Spine Disorders |
| Neurologic | Dementia |
| | Epilepsy/Convulsions |
| | Multiple Sclerosis |
| | Parkinson's Disease |
| | Peripheral Nerve Disorders |
| | Spinal Cord Injury or Paralysis |
| | Traumatic Brain Injury |
| Other | Chronic Pain Syndromes |
| | Tobacco Use Disorder |
| Renal/Urinary | Renal Failure or Nephropathy |
| Respiratory | Allergic and Other Chronic Sinusitis/Rhinitis |
| | Asthma |
| | Chronic Obstructive Pulmonary Disease |

**eTable 1: Patient characteristics, care fragmentation scores and outcomes for overall high-risk patient cohort (n=130, 704)**

| | **High-Risk Patients (n= 130,704)** |
|---|---|
| *CAN 1 year hospitalization* | 0.36 (0.15) |
| 90th | 62, 930 (48.2%) |
| 95th | 12, 902 (9.9%) |
| 96th | 13, 212 (10.1%) |
| 97th | 13, 437 (10.3%) |
| 98th | 13, 999 (10.7%) |
| 99th | 14, 224 (10.9%) |
| *Gender* | |
| FEMALE | 3035 (2.3%) |
| MALE | 127669 (97.7%) |
| *Age* | 73.74 (7.98) |
| *Marital Status* | |
| Not Married | 69334 (53.0%) |
| Married | 60846 (46.6%) |
| Missing | 524 (0.4%) |
| *VA Priority Group* | |
| 1. Priority 7+8 | 11436 (8.7%) |
| 2. Priority 5 (low income) | 33021 (25.3%) |
| 3. Priority 1+4 (high disability) | 69879 (53.5%) |
| 4. Priority 2+3+6 (low/moderate disability) | 16368 (12.5%) |
| *Race/Ethnicity* | |
| Black | 20292 (15.5%) |
| Hispanic | 4793 (3.7%) |
| Other | 2192 (1.7%) |
| Unknown | 4407 (3.4%) |
| White | 99020 (75.8%) |
| *Urban/Rural* | |
| Urban | 81784 (62.6%) |
| Rural/Highly Rural | 48919 (37.4%) |
| *Number Chronic Conditions, mean (SD)* | 9.03 (4.01) |
| *Any mental health condition* | 63995 (49.0%) |
| *Selected Chronic Conditions* | |
| Hypertension | 109988 (84.2%) |
| Coronary Artery Disease | 60811 (46.5%) |
| Heart Failure | 44715 (34.2%) |
| Diabetes Mellitus | 68043 (52.1%) |
| Depression | 40542 (31.0%) |
| PTSD | 23843 (18.2%) |
| Anxiety Disorders | 18302 (14.0%) |
| Dementia | 17498 (13.4%) |
| Renal Failure or Nephropathy | 46811 (35.8%) |
| Chronic Obstructive Pulmonary Disease | 48437 (37.1%) |
| *Care Fragmentation Indices* | |
| Number Providers, mean (SD) | 7.06 (3.72) |
| Number Providers, median (IQR) | 6.00 (4.00, 9.00) |
| UPC*, mean (SD) | 0.36 (0.16) |
| UPC*, median (IQR) | 0.33 (0.25, 0.44) |
| *Outcomes in FY15* | |

© 2022 Edwards ST et al. *JAMA Network Open.*

| | |
|---|---|
| Hospitalization | 60262 (46.1%) |
| Deceased | 18564 (14.2%) |
| Hospitalized or Died | 65151 (49.8%) |
| ACSC Hospitalization | 19020 (14.6%) |
| Any ED Visit | 88350 (67.6%) |
| ED visits, mean (SD) | 2.11 (2.84) |

* UPC Usual Provider of Care, the proportion of care with the most frequently seen provider, ranges from 0 to 1 where 1 indicates that all visits occur with one provider.

© 2022 Edwards ST et al. *JAMA Network Open.*

**eTable 2 . Patient Characteristics by UPC Tertile**

| | UPC Tertile | | | |
|---|---|---|---|---|
| | Low (0.08-0.40) | Medium (0.41 - 0.66) | High (0.67 - 1) | p-value |
| N | 3155 | 2995 | 2758 | |
| *CAN 1 year hospitalization probability* | 0.43 (0.17) | 0.40 (0.17) | 0.39 (0.16) | <0.001 |
| *CAN percentiles* | | | | <0.001 |
| 90th | 1071 (33.9%) | 1137 (38.0%) | 1167 (42.3%) | |
| 95th | 276 (8.7%) | 286 (9.5%) | 236 (8.6%) | |
| 96th | 314 (10.0%) | 284 (9.5%) | 293 (10.6%) | |
| 97th | 328 (10.4%) | 354 (11.8%) | 304 (11.0%) | |
| 98th | 483 (15.3%) | 414 (13.8%) | 352 (12.8%) | |
| 99th | 683 (21.6%) | 520 (17.4%) | 406 (14.7%) | |
| *Gender* | | | | 0.031 |
| FEMALE | 93 (2.9%) | 95 (3.2%) | 114 (4.1%) | |
| MALE | 3062 (97.1%) | 2900 (96.8%) | 2644 (95.9%) | |
| *Age* | 78.75 (8.87) | 79.93 (8.95) | 81.49 (9.04) | <0.001 |
| *Marital Status* | | | | <0.001 |
| Not Married | 1698 (53.8%) | 1735 (57.9%) | 1658 (60.1%) | |
| Married | 1437 (45.5%) | 1243 (41.5%) | 1081 (39.2%) | |
| Missing | 20 (0.6%) | 17 (0.6%) | 19 (0.7%) | |
| *VA Priority Group* | | | | <0.001 |
| 1. Priority 7+8 | 160 (5.1%) | 134 (4.5%) | 112 (4.1%) | |
| 2. Priority 5 (low income) | 567 (18.0%) | 522 (17.4%) | 393 (14.2%) | |
| 3. Priority 1+4 (high disability) | 2037 (64.6%) | 1984 (66.2%) | 1949 (70.7%) | |
| 4. Priority 2+3+6 (low/moderate disability) | 391 (12.4%) | 355 (11.9%) | 304 (11.0%) | |
| *Race/Ethnicity* | | | | <0.001 |
| Black | 547 (17.3%) | 501 (16.7%) | 514 (18.6%) | |
| Hispanic | 71 (2.3%) | 90 (3.0%) | 88 (3.2%) | |
| Other | 51 (1.6%) | 50 (1.7%) | 56 (2.0%) | |
| Unknown | 134 (4.2%) | 130 (4.3%) | 177 (6.4%) | |
| White | 2352 (74.5%) | 2224 (74.3%) | 1923 (69.7%) | |
| *Urban/Rural* | | | | 0.041 |
| Urban | 2208 (70.0%) | 2007 (67.0%) | 1880 (68.2%) | |
| Rural/Highly Rural | 947 (30.0%) | 988 (33.0%) | 878 (31.8%) | |
| *Number Chronic Conditions, mean (SD)* | 11.73 (3.96) | 11.34 (3.87) | 10.62 (3.68) | <0.001 |
| *Any mental health condition* | 1917 (60.8%) | 1783 (59.5%) | 1737 (63.0%) | 0.026 |
| *Selected Chronic Conditions* | | | | |
| Hypertension | 2931 (92.9%) | 2751 (91.9%) | 2492 (90.4%) | 0.002 |
| Coronary Artery Disease | 1890 (59.9%) | 1678 (56.0%) | 1405 (50.9%) | <0.001 |
| Heart Failure | 1671 (53.0%) | 1508 (50.4%) | 1252 (45.4%) | <0.001 |
| Diabetes Mellitus | 1749 (55.4%) | 1695 (56.6%) | 1392 (50.5%) | <0.001 |
| Depression | 1423 (45.1%) | 1301 (43.4%) | 1305 (47.3%) | 0.013 |
| PTSD | 489 (15.5%) | 446 (14.9%) | 311 (11.3%) | <0.001 |
| Anxiety Disorders | 647 (20.5%) | 591 (19.7%) | 546 (19.8%) | 0.70 |
| Dementia | 957 (30.3%) | 1147 (38.3%) | 1353 (49.1%) | <0.001 |
| Renal Failure or Nephropathy | 1571 (49.8%) | 1400 (46.7%) | 1130 (41.0%) | <0.001 |
| Chronic Obstructive Pulmonary Disease | 1530 (48.5%) | 1421 (47.4%) | 1214 (44.0%) | 0.002 |

© 2022 Edwards ST et al. *JAMA Network Open.*

**eTable 3. Full logistic regression models of relationship between outpatient care fragmentation in FY14 and FY15 hospitalization or ED Visit for HBPC patients (n=8,852)**

| | ACSC Hospitalization | | | Any ED Visit | | |
|---|---|---|---|---|---|---|
| | **Providers** | **UPC** | **UPC as Tertile** | **Providers** | **UPC** | **UPC as Tertile** |
| **Number of Providers** | 1.04 (1.02 - 1.06) | | | 1.05 (1.03 - 1.07) | | |
| **UPC** | | 0.62 (0.49 - 0.78) | | | 0.61 (0.49 - 0.75) | |
| **UPC in tertiles (ref: low (0.08 - 0.40))** | | | | | | |
| Medium (>0.4 - 0.67) | | | 0.94 (0.83 - 1.06) | | | 0.85 (0.76 - 0.96) |
| High (>0.67 - 1) | | | 0.77 (0.67 - 0.88) | | | 0.78 (0.68 - 0.88) |
| **Age** | 1.01 (1.01 - 1.02) | 1.01 (1.01 - 1.02) | 1.01 (1.01 - 1.02) | 1.02 (1.01 - 1.02) | 1.02 (1.01 - 1.02) | 1.02 (1.01 - 1.02) |
| **Gender: Male** | 1.08 (0.81 - 1.45) | 1.08 (0.80 - 1.44) | 1.08 (0.81 - 1.45) | 1.26 (0.97 - 1.63) | 1.25 (0.96 - 1.61) | 1.25 (0.97 - 1.62) |
| **Married** | 1.09 (0.98 - 1.21) | 1.09 (0.98 - 1.21) | 1.09 (0.98 - 1.21) | 0.95 (0.86 - 1.05) | 0.95 (0.86 - 1.05) | 0.96 (0.87 - 1.05) |
| **Race/Ethnicity (Ref: Black/African American)** | | | | | | |
| White | 1.11 (0.96 - 1.28) | 1.10 (0.96 - 1.27) | 1.10 (0.96 - 1.27) | 0.95 (0.83 - 1.08) | 0.94 (0.83 - 1.07) | 0.95 (0.83 - 1.08) |
| Hispanic/Latino | 1.15 (0.82 - 1.59) | 1.15 (0.82 - 1.60) | 1.14 (0.82 - 1.59) | 1.01 (0.74 - 1.38) | 1.01 (0.74 - 1.38) | 1.01 (0.74 - 1.38) |
| Other | 1.16 (0.77 - 1.73) | 1.15 (0.77 - 1.73) | 1.15 (0.77 - 1.73) | 0.99 (0.68 - 1.44) | 0.99 (0.68 - 1.44) | 0.99 (0.68 - 1.44) |
| Unknown | 1.13 (0.87 - 1.48) | 1.13 (0.87 - 1.47) | 1.13 (0.87 - 1.47) | 0.85 (0.67 - 1.08) | 0.85 (0.67 - 1.08) | 0.84 (0.66 - 1.07) |
| **Geographic Location: Rural** | 0.98 (0.87 - 1.10) | 0.98 (0.87 - 1.09) | 0.97 (0.87 - 1.09) | 0.89 (0.80 - 0.99) | 0.88 (0.80 - 0.98) | 0.88 (0.80 - 0.98) |
| **VA Priority (Ref: 7 & 8)** | | | | | | |
| Priority 5 (low income) | 1.17 (0.90 - 1.54) | 1.18 (0.90 - 1.54) | 1.17 (0.90 - 1.54) | 1.11 (0.87 - 1.42) | 1.11 (0.87 - 1.42) | 1.11 (0.87 - 1.42) |
| Priority 1 & 4 (high disability) | 0.99 (0.77 - 1.27) | 1.00 (0.78 - 1.28) | 0.99 (0.77 - 1.27) | 1.18 (0.94 - 1.48) | 1.19 (0.95 - 1.49) | 1.18 (0.94 - 1.48) |
| Priority 2 & 3 & 6 (low - moderate disability) | 1.01 (0.76 - 1.33) | 1.01 (0.76 - 1.33) | 1.00 (0.76 - 1.33) | 1.12 (0.87 - 1.45) | 1.12 (0.87 - 1.45) | 1.12 (0.87 - 1.45) |
| **Number of chronic conditions** | 1.09 (1.08 - 1.11) | 1.10 (1.08 - 1.11) | 1.10 (1.08 - 1.11) | 1.07 (1.06 - 1.09) | 1.07 (1.06 - 1.09) | 1.07 (1.06 - 1.09) |
| **MH condition** | 0.86 (0.76 - 0.96) | 0.86 (0.76 - 0.96) | 0.86 (0.76 - 0.96) | 0.99 (0.89 - 1.11) | 0.99 (0.89 - 1.10) | 0.99 (0.89 - 1.10) |
| **Total outpatient visits** | 0.99 (0.99 - 1.00) | 1.00 (1.00 - 1.01) | 1.00 (1.00 - 1.01) | 1.00 (1.00 - 1.01) | 1.01 (1.01 - 1.02) | 1.01 (1.01 - 1.02) |
| **Any Medicare visit** | 0.87 (0.77 - 0.97) | 0.84 (0.75 - 0.95) | 0.85 (0.76 - 0.96) | 0.93 (0.83 - 1.04) | 0.92 (0.82 - 1.03) | 0.94 (0.84 - 1.05) |
| **HBPC encounters in Q4** | 1.01 (1.00 - 1.02) | 1.01 (1.00 - 1.02) | 1.01 (1.00 - 1.02) | 1.01 (1.00 - 1.02) | 1.01 (1.00 - 1.02) | 1.01 (1.00 - 1.02) |
| **Intercept** | 0.03 (0.02 - 0.06) | 0.04 (0.02 - 0.08) | 0.04 (0.02 - 0.07) | 0.20 (0.11 - 0.37) | 0.30 (0.16 - 0.55) | 0.27 (0.15 - 0.49) |

Odds ratio and 95% confidence interval.

© 2022 Edwards ST et al. *JAMA Network Open.*

**eTable 4. Sensitivity Analysis: Full logistic regression models of relationship between outpatient care fragmentation in FY14 and FY15 hospitalization or ED Visit for HBPC patients, excluding those who died in FY15 (n=6,464)**

| | ACSC Hospitalization | | | Any ED Visit | | |
|---|---|---|---|---|---|---|
| | **Providers** | **UPC** | **UPC as Tertile** | **Providers** | **UPC** | **UPC as Tertile** |
| **Number of Providers** | 1.04 (1.02 - 1.06) | | | 1.06 (1.03 - 1.08) | | |
| **UPC** | | 0.65 (0.49 - 0.87) | | | 0.59 (0.47 - 0.76) | |
| **UPC in tertiles (ref: low (0.08 - 0.40))** | | | | | | |
| Medium (>0.4 - 0.67) | | | 0.95 (0.82 - 1.1) | | | 0.84 (0.74 - 0.96) |
| High (>0.67 - 1) | | | 0.79 (0.67 - 0.94) | | | 0.77 (0.67 - 0.89) |
| **Age** | 1.01 (1.01 - 1.02) | 1.01 (1.01 - 1.02) | 1.01 (1.01 - 1.02) | 1.02 (1.01 - 1.02) | 1.02 (1.01 - 1.02) | 1.02 (1.01 - 1.02) |
| **Gender: Male** | 1.12 (0.80 - 1.58) | 1.11 (0.79 - 1.56) | 1.11 (0.79 - 1.56) | 1.19 (0.89 - 1.58) | 1.18 (0.89 - 1.57) | 1.18 (0.89 - 1.57) |
| **Married** | 1.07 (0.94 - 1.21) | 1.07 (0.94 - 1.22) | 1.07 (0.94 - 1.22) | 0.98 (0.87 - 1.10) | 0.99 (0.88 - 1.10) | 0.99 (0.88 - 1.10) |
| **Race/Ethnicity (Ref: Black/African American)** | | | | | | |
| White | 1.08 (0.91 - 1.28) | 1.07 (0.90 - 1.27) | 1.07 (0.91 - 1.27) | 0.94 (0.81 - 1.08) | 0.93 (0.80 - 1.08) | 0.93 (0.81 - 1.08) |
| Hispanic/Latino | 0.95 (0.63 - 1.44) | 0.95 (0.63 - 1.44) | 0.95 (0.63 - 1.44) | 1.03 (0.72 - 1.47) | 1.03 (0.73 - 1.48) | 1.03 (0.72 - 1.47) |
| Other | 1.17 (0.74 - 1.84) | 1.17 (0.74 - 1.84) | 1.17 (0.74 - 1.84) | 0.96 (0.64 - 1.43) | 0.96 (0.64 - 1.43) | 0.96 (0.64 - 1.43) |
| Unknown | 1.07 (0.77 - 1.48) | 1.06 (0.76 - 1.47) | 1.06 (0.76 - 1.48) | 0.84 (0.63 - 1.11) | 0.83 (0.63 - 1.10) | 0.83 (0.62 - 1.10) |
| **Geographic Location: Rural** | 0.97 (0.85 - 1.11) | 0.97 (0.84 - 1.11) | 0.96 (0.84 - 1.11) | 0.93 (0.83 - 1.05) | 0.93 (0.82 - 1.04) | 0.92 (0.82 - 1.04) |
| **VA Priority (Ref: 7 & 8)** | | | | | | |
| Priority 5 (low income) | 1.23 (0.88 - 1.73) | 1.24 (0.88 - 1.74) | 1.23 (0.88 - 1.73) | 1.13 (0.85 - 1.50) | 1.13 (0.85 - 1.50) | 1.13 (0.85 - 1.50) |
| Priority 1 & 4 (high disability) | 1.12 (0.82 - 1.54) | 1.13 (0.82 - 1.54) | 1.12 (0.82 - 1.54) | 1.28 (0.99 - 1.66) | 1.28 (0.99 - 1.66) | 1.28 (0.99 - 1.66) |
| Priority 2 & 3 & 6 (low - moderate disability) | 1.23 (0.86 - 1.75) | 1.23 (0.86 - 1.75) | 1.23 (0.86 - 1.75) | 1.22 (0.91 - 1.65) | 1.22 (0.90 - 1.64) | 1.22 (0.91 - 1.65) |
| **Number of chronic conditions** | 1.11 (1.09 - 1.13) | 1.11 (1.09 - 1.13) | 1.11 (1.09 - 1.13) | 1.08 (1.06 - 1.10) | 1.08 (1.06 - 1.10) | 1.08 (1.06 - 1.10) |
| **MH condition** | 0.85 (0.74 - 0.98) | 0.85 (0.73 - 0.97) | 0.84 (0.73 - 0.97) | 1.03 (0.91 - 1.16) | 1.02 (0.90 - 1.15) | 1.01 (0.90 - 1.15) |
| **Total outpatient visits** | 0.99 (0.99 - 1.00) | 1.00 (0.99 - 1.01) | 1.00 (1.00 - 1.01) | 1.00 (0.99 - 1.01) | 1.01 (1.00 - 1.02) | 1.01 (1.00 - 1.02) |
| **Any Medicare visit** | 0.92 (0.80 - 1.06) | 0.91 (0.79 - 1.05) | 0.92 (0.79 - 1.06) | 0.93 (0.82 - 1.06) | 0.92 (0.81 - 1.05) | 0.94 (0.82 - 1.07) |
| **HBPC encounters in Q4** | 1.01 (1.00 - 1.02) | 1.01 (1.00 - 1.02) | 1.00 (0.99 - 1.02) | 1.01 (1.00 - 1.02) | 1.01 (1.00 - 1.02) | 1.01 (1.00 - 1.02) |
| **Intercept** | 0.02 (0.01 - 0.04) | 0.02 (0.01 - 0.05) | 0.02 (0.01 - 0.05) | 0.15 (0.07 - 0.30) | 0.23 (0.12 - 0.47) | 0.21 (0.10 - 0.42) |

Odds ratio and 95% confidence interval.

© 2022 Edwards ST et al. *JAMA Network Open.*

**eTable 5. Sensitivity Analysis: Negative binomial regression models of relationship between outpatient care fragmentation in FY14 and number of FY15 ED Visits for HBPC patients (n=8,852)**

| | Count of ED Visits | | |
|---|---|---|---|
| | **Providers** | **UPC** | **UPC as Tertile** |
| **Number of Providers** | 1.04 (1.03 - 1.05) | | |
| **UPC** | | 0.70 (0.63 - 0.78) | |
| **UPC in tertiles (ref: low (0.08 - 0.40))** | | | |
| Medium (>0.4 - 0.67) | | | 0.93 (0.88 - 0.98) |
| High (>0.67 - 1) | | | 0.83 (0.78 - 0.88) |
| **Age** | 1.00 (1.00 - 1.01) | 1.00 (1.00 - 1.01) | 1.00 (1.00 - 1.01) |
| **Gender: Male** | 1.14 (1.00 - 1.30) | 1.13 (0.99 - 1.29) | 1.14 (1.00 - 1.30) |
| **Married** | 0.98 (0.93 - 1.03) | 0.99 (0.94 - 1.04) | 0.99 (0.94 - 1.04) |
| **Race/Ethnicity (Ref: Black/African American)** | | | |
| White | 0.92 (0.87 - 0.98) | 0.92 (0.87 - 0.98) | 0.92 (0.87 - 0.98) |
| Hispanic/Latino | 1.02 (0.88 - 1.19) | 1.02 (0.88 - 1.19) | 1.02 (0.88 - 1.19) |
| Other | 0.99 (0.83 - 1.19) | 0.99 (0.83 - 1.19) | 1.00 (0.83 - 1.19) |
| Unknown | 0.82 (0.73 - 0.93) | 0.82 (0.73 - 0.93) | 0.82 (0.73 - 0.93) |
| **Geographic Location: Rural** | 0.96 (0.91 - 1.01) | 0.95 (0.90 - 1.00) | 0.95 (0.90 - 1.00) |
| **VA Priority (Ref: 7 & 8)** | | | |
| Priority 5 (low income) | 1.18 (1.04 - 1.34) | 1.19 (1.05 - 1.35) | 1.19 (1.05 - 1.35) |
| Priority 1 & 4 (high disability) | 1.10 (0.98 - 1.24) | 1.11 (0.99 - 1.24) | 1.10 (0.99 - 1.24) |
| Priority 2 & 3 & 6 (low - moderate disability) | 1.13 (0.99 - 1.28) | 1.13 (0.99 - 1.29) | 1.13 (0.99 - 1.28) |
| **Number of chronic conditions** | 1.06 (1.05 - 1.06) | 1.06 (1.05 - 1.06) | 1.06 (1.05 - 1.07) |
| **MH condition** | 0.99 (0.94 - 1.04) | 0.99 (0.94 - 1.04) | 0.99 (0.94 - 1.04) |
| **Total outpatient visits** | 1.00 (1.00 - 1.00) | 1.01 (1.00 - 1.01) | 1.01 (1.00 - 1.01) |
| **Any Medicare visit** | 0.91 (0.87 - 0.96) | 0.91 (0.86 - 0.96) | 0.92 (0.87 - 0.97) |
| **HBPC encounters in Q4** | 1.01 (1.00 - 1.01) | 1.00 (1.00 - 1.01) | 1.00 (1.00 - 1.01) |
| **Intercept** | 0.62 (0.46 - 0.84) | 0.84 (0.62 - 1.14) | 0.77 (0.57 - 1.04) |

Incidence Rate Ratio and 95% confidence intervals.
The likelihood ratio test for over-dispersion was significant for each of the models.

© 2022 Edwards ST et al. *JAMA Network Open.*

**eTable 6. Sensitivity Analysis: Adjusted relationship between UPC in FY14 and FY15 hospitalization or ED Visit for HBPC patients, excluding those with UPC = 1 (n=8,247)**

| | ACSC Hospitalization | | Any ED Visit | |
|---|---|---|---|---|
| | **UPC** | **UPC as Tertile** | **UPC** | **UPC as Tertile** |
| **UPC** | 0.63 (0.49 - 0.82) | | 0.59 (0.46 - 0.75) | |
| **UPC in tertiles (ref: low (0.08 - 0.40))** | | | | |
| Medium (>0.4 - 0.67) | | 0.94 (0.83 - 1.06) | | 0.85 (0.76 - 0.96) |
| High (>0.67 - 1) | | 0.79 (0.68 - 0.91) | | 0.79 (0.69 - 0.90) |
| **Age** | 1.01 (1.01 - 1.02) | 1.01 (1.01 - 1.02) | 1.02 (1.01 - 1.02) | 1.02 (1.01 - 1.02) |
| **Gender: Male** | 1.08 (0.79 - 1.46) | 1.08 (0.80 - 1.46) | 1.20 (0.91 - 1.57) | 1.20 (0.91 - 1.58) |
| **Married** | 1.08 (0.97 - 1.20) | 1.08 (0.97 - 1.20) | 0.96 (0.86 - 1.06) | 0.96 (0.87 - 1.06) |
| **Race/Ethnicity (Ref: Black/African American)** | | | | |
| White | 1.11 (0.96 - 1.29) | 1.11 (0.96 - 1.29) | 0.95 (0.83 - 1.09) | 0.96 (0.84 - 1.10) |
| Hispanic/Latino | 1.19 (0.85 - 1.68) | 1.19 (0.85 - 1.68) | 1.09 (0.78 - 1.52) | 1.09 (0.78 - 1.52) |
| Other | 1.12 (0.73 - 1.71) | 1.12 (0.73 - 1.71) | 1.01 (0.69 - 1.50) | 1.01 (0.69 - 1.50) |
| Unknown | 1.10 (0.84 - 1.46) | 1.10 (0.84 - 1.46) | 0.82 (0.64 - 1.06) | 0.82 (0.64 - 1.06) |
| **Geographic Location: Rural** | 0.97 (0.86 - 1.09) | 0.96 (0.86 - 1.08) | 0.88 (0.79 - 0.98) | 0.88 (0.79 - 0.98) |
| **VA Priority (Ref: 7 & 8)** | | | | |
| Priority 5 (low income) | 1.15 (0.88 - 1.52) | 1.15 (0.87 - 1.52) | 1.10 (0.85 - 1.42) | 1.10 (0.85 - 1.42) |
| Priority 1 & 4 (high disability) | 0.97 (0.76 - 1.26) | 0.97 (0.75 - 1.25) | 1.19 (0.94 - 1.50) | 1.19 (0.94 - 1.50) |
| Priority 2 & 3 & 6 (low - moderate disability) | 1.01 (0.76 - 1.35) | 1.01 (0.76 - 1.35) | 1.16 (0.89 - 1.52) | 1.16 (0.89 - 1.52) |
| **Number of chronic conditions** | 1.10 (1.08 - 1.12) | 1.10 (1.08 - 1.12) | 1.07 (1.06 - 1.09) | 1.07 (1.06 - 1.09) |
| **MH condition** | 0.87 (0.77 - 0.98) | 0.87 (0.77 - 0.98) | 1.01 (0.90 - 1.13) | 1.01 (0.90 - 1.12) |
| **Total outpatient visits** | 1.00 (1.00 - 1.01) | 1.00 (1.00 - 1.01) | 1.01 (1.00 - 1.02) | 1.01 (1.00 - 1.02) |
| **Any Medicare visit** | 0.84 (0.75 - 0.95) | 0.85 (0.75 - 0.95) | 0.92 (0.82 - 1.03) | 0.93 (0.83 - 1.04) |
| **HBPC encounters in Q4** | 1.01 (1.00 - 1.01) | 1.01 (1.00 - 1.01) | 1.01 (1.00 - 1.02) | 1.01 (1.00 - 1.02) |
| **Intercept** | 0.04 (0.02 - 0.08) | 0.04 (0.02 - 0.08) | 0.31 (0.17 - 0.59) | 0.28 (0.15 - 0.53) |

Odds ratio and 95% confidence interval.

© 2022 Edwards ST et al. *JAMA Network Open.*

**eTable 7. Adjusted relationship between outpatient care fragmentation in FY14 and FY15 hospitalization or ED Visit for HBPC patients, all providers considered unique (no accounting for HBPC team)**

| Outcome | Fragmentation Measure | Odds Ratio (95% Confidence Interval) |
|---|---|---|
| Any ACSC Hospitalization | Provider count | 1.03 (1.01-1.05) |
| | UPC (continuous) | 0.72 (0.52 – 1.00) |
| Any ED Visit | Provider count | 1.05 (1.03-1.07) |
| | UPC (continuous) | 0.62 (0.46 - 0.83) |

Note: UPC presented continuous as tertiles were calculated for HBPC-specific UPC only.

© 2022 Edwards ST et al. *JAMA Network Open.*
